# Improving general practice appointment booking processes: bureaucratic justice and patient-centred reforms

**DOI:** 10.3399/BJGP.2025.0230

**Published:** 2025-07-22

**Authors:** Aisling Ryan, Jed Meers, Joe Tomlinson, Lina Gega, Andrew S Moriarty, Laura Jefferson

**Affiliations:** 1 York Law School, University of York, York, UK; 2 Hull York Medical School, Heslington, England, UK; 3 Department of Health Sciences, University of York, York, UK

## Introduction

‘Bureaucracy’ is a loaded term in general practice. In October 2024, UK Secretary of State for Health and Social Care, Wes Streeting, announced a ‘red tape challenge’ to cut down on general practice bureaucracy at the RCGP annual conference. But is cutting down on bureaucracy the right starting point to bring about positive change in general practice administration?

New administrative processes are usually a response to a particular problem or designed to implement a particular policy goal and are crucial to how patients perceive and experience bureaucracy in a particular area of administration. The ‘Modern General Practice’ model has been introduced and incentivised as a proposed solution to demand outstripping capacity and increased complexity. Hybrid models implemented over the COVID-19 pandemic have persisted and these have introduced new administrative processes for patients when requesting appointments such as online triage forms, the use of different digital applications, and increased navigation towards remote and non-GP appointments.

Despite the well-intentioned motivations behind such developments, Payne *et al*’s recent study found that *‘*[s]*ystems designed to increase efficiency have introduced new forms of inefficiency and have compromised other quality domains such as accessibility, patient-centredness, and equity’.* ^1^ In a related editorial, Lawson memorably described the ‘enshittification’ of general practice in the UK, referring in large part to the diminished experience for patients, GPs, and their primary care teams that can result from such initiatives.^2^ We urgently need to pay closer attention to process qualities, and to develop a smarter and more considered approach to general practice administration. Such an approach should be evidence based and co-produced with stakeholders to develop frameworks to refine administrative processes. We argue that ‘bureaucratic justice’ — a field of study that explores administrative interactions between individuals and public services (also referred to as ‘administrative justice’) — provides a timely and empirically grounded way of researching and reforming patient-facing general practice administration.

## The potential of bureaucratic justice for general practice appointment booking processes

The processes through which GP appointments are booked constitute one of the largest and most important interfaces both within the NHS and the public sector overall. An average of 1.42 million GP appointments are delivered every working day in England.^3^ Existing research on the process side of GP appointments tends to focus on the causes of missed appointments^4^ or the impacts of missed appointments on health services.^5^ Beyond this, there is remarkably little evidence on the bureaucratic aspects of GP appointment booking processes. A recent scoping review suggests that existing research on access to GP appointments focuses on managing demand and reducing GP workload pressures from a practice perspective rather than aiming to understand and improve processes from a patient perspective.^6^ Understanding administrative process reform as a source of justice, as well as efficiency, is vital — and not mutually exclusive. Indeed, more just processes can also be more efficient.

Bureaucratic justice is a field of study that is concerned with everyday experiences of public services and how administrative systems are organised.^7^ In broad terms, the field is concerned with what is ‘just’ or ‘fair’ in interactions between individuals and the state. Much of this body of work has focused on certain areas of administration including social security^8^ and immigration.^9^ Bureaucratic justice research has expanded in recent years to more meaningfully capture and engage with the subjective views of system users and positioning these views as central to understanding how systems operate and what can improve these systems.^10^

There has been limited research carried out to date at the intersection of bureaucratic justice and health care, but the few existing studies have found that people’s perception of fair treatment in encounters with health care are important for improving patient outcomes. Perception of fair process has been linked to greater adherence to GP’s advice^11^ and improved effectiveness of weight-loss programmes.^12^ In order to address this gap in knowledge, the Administrative Fairness Lab and Institute of Mental Health Research at York have been working together across disciplinary boundaries to develop a research agenda on bureaucratic justice and health care. We have spoken to members of the public through Patient and Public Involvement (PPI) focus groups, alongside engagement with GPs and GP administrators. This work has indicated that GP appointment booking processes can be sites of mass injustice. This injustice is not spread equally across society; some of the stakeholder engagement points to how there is a greater burden on people who face societal barriers to access such as digital exclusion, and on people experiencing poor health or multimorbidities.

**Figure 1.**
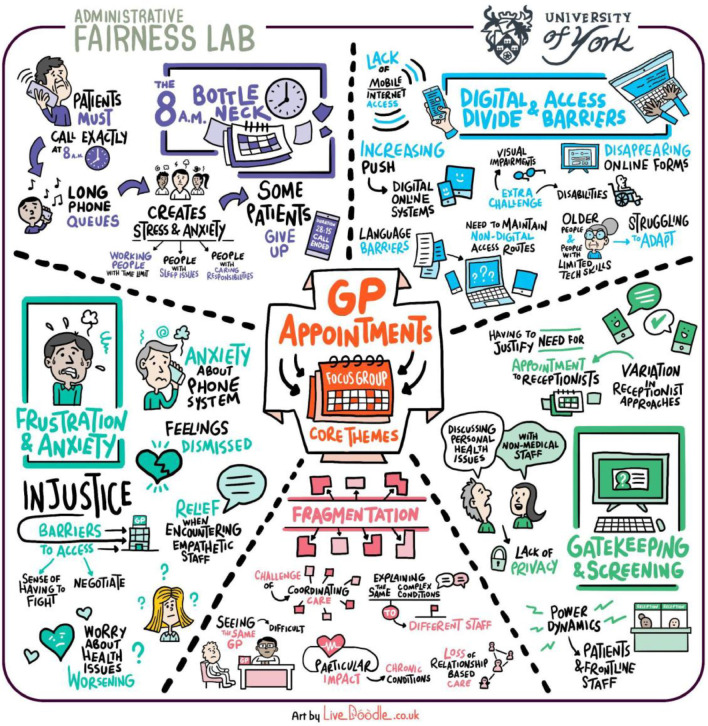
Infographic illustrating issues discussed at PPI focus groups. PPI = Patient and Public Involvement.

In our PPI focus groups, people spoke about having to ‘plead’ with GP receptionists for appointments, going ‘around in circles’ trying to secure an appointment, or borrowing money to use private health care because of unavailability of appointments through the NHS. The PPI focus groups revealed some of the complex and intersecting societal phenomena that relate to the lived experience of GP appointment booking processes (Figure 1).

There is a strong indication that the public’s experiences are getting worse: *‘There used to be quite a good online system where you could book it* [an appointment] *yourself. Now, the form you have to fill in is pages and pages long’* (Public involvement representative). Good experiences of booking a GP appointment dropped to 54.4% in 2023 — down from 56.2% in 2022, and 70.6% in 2021.^13^ Reduced workforce capacity because of GPs leaving or changing their hours, coupled with increased patient demand has led to a sector in crisis. With 80% of practices currently taking some form of collection action – for example, through capping appointments to 25 a day as part of the BMA ‘practice survival toolkit’,^14^ systems rationing access to appointments are under unprecedented demand.

Research from the field of procedural justice indicates that there are tangible benefits from making systems more just for users: *‘there is a great deal of evidence that people are motivated by their justice judgments’*.^15^ Tyler’s work demonstrates that perceptions of trust and legitimacy in public processes are more likely to achieve desired outcomes from a public policy perspective.^15^ There is good ground to think that, if work is done to make GP appointment booking processes more just, there will be benefits for patients and practices. Such benefits might include increased willingness to book, attend, or wait for an appointment.

## Health inequalities and digital exclusion

The King’s Fund highlights how poor administrative processes can exacerbate health inequalities and notes that the required response is not necessarily less administration — instead there is a need to focus on ‘better admin’.^16^ Administrative processes for booking GP appointments are now so bad that patients actively avoid engaging with the systems that are, in theory, designed to help them: ‘*The process puts you off trying to see a doctor*.’^17^

Gkiouleka *et al* note that general practice can contribute to reducing health inequalities, but there is limited research on the types of interventions that are proven to have this effect.^18^ They also note that most of the existing evidence base flows from *‘controlled trials that do not address the effect of the social determinants of health’*. Bureaucratic injustice can be understood as a social determinant of health — part of the social, political, and economic circumstances that influence health across the life course — with the design and operationalisation of public services limiting opportunity and contributing to health inequity.^19^

We need more evidence to understand the role that administrative processes play in how patients interact (or don’t interact) with general practice. The administrative burden placed on people who turn to the state for support can contribute to disengagement.^20^ People experience administrative processes in different ways, and because of different local ecosystems there are different barriers to access. There are known administrative barriers such as the requirement for a fixed address that contributes to difficulties for people experiencing homelessness accessing primary care, but there are likely many unknown administrative barriers yet to be captured or fully unravelled. Particular attention must be paid to how underserved or marginalised groups are impacted by bureaucratic injustice or barriers to access.^21^

Our ageing society and the drive towards digital needs further interrogation: *‘The assumption that everyone has a smart phone. I do not.’* ^17^ The digitalisation of certain administrative processes relating to booking an appointment arose in our PPI focus groups, with form-design and temporality playing a role in how patients interact with general practice.

Yet GPs are also bogged down by the burden that is now in-built into the system through private sector providers, the GP contract, and wider socio-technical problems. Barnard *et al*’s ethnographic study illuminates the ‘hidden work’ carried out by GPs, whereby carrying out administrative work outside of normal working hours is normalised, exacerbating workload pressure.^22^ The processes are often failing everyone.

## Process qualities and future research

**Figure 2.**
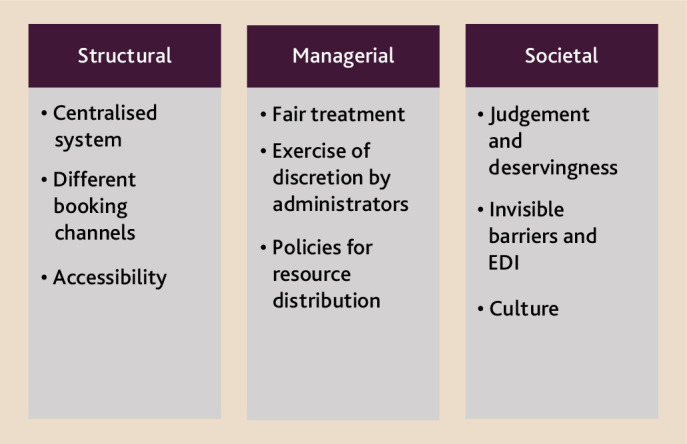
Identifying potential future research inquiries on the different aspects of GP appointment booking processes. EDI = equality, diversity, and inclusion.

In developing research on bureaucratic justice and GP appointment booking processes, existing bureaucratic justice research and our initial stakeholder engagement work points to three process-based investigations required to understand subjective perceptions on GP appointment booking processes (Figure 2).

A lived experience-led understanding of the structural, managerial, and societal administrative aspects of GP appointment booking processes has the potential to develop rich and novel knowledge on one of the biggest administrative processes of the state, and to theorise, develop, and evaluate ways of booking GP appointments. Patients have a complex set of values and preferences informing their perceptions on GP appointment booking processes and do not always hold speed of access to an appointment as the most important factor.^23^ New models of bureaucratic justice are being developed in other areas, such as social security.^24^ Because of the contextual nature of public administration, it is likely that different process qualities will be deduced from data in different contexts.

## Conclusion

Priority-setting PPI work and research from the field of bureaucratic justice suggests that, to secure the highest satisfaction level and the best possible long-term outcomes for patients, we must not just strive for GP appointment booking processes to be perceived as efficient but also for them to be perceived as legitimate and just. Gathering data on such perceptions will require an interdisciplinary mixed-methods study, with the views of patients, GPs, administrators, system designers, and policymakers all instrumental in developing process interventions to improve experiences and perceptions of GP appointment booking processes.

## Public involvement

Public involvement representatives consented to anonymised data being used in written outputs. A total of seven online PPI focus groups took place in October 2023 and June 2024, facilitated by Dr Jed Meers and Dr Aisling Ryan with organisational coordination from Leigh Kristensen, Sparks Coordinator.
